# Spatial Distribution and Migration Characteristic of Forchlorfenuron in Oriental Melon Fruit by Matrix-Assisted Laser Desorption/Ionization Mass Spectrometry Imaging

**DOI:** 10.3390/foods12152858

**Published:** 2023-07-27

**Authors:** Qi Wang, Xiaohui Li, Hongping Wang, Simeng Li, Chen Zhang, Xueying Chen, Jing Dong, Hua Shao, Jing Wang, Fen Jin

**Affiliations:** 1 Key Laboratory of Agro-Product Quality and Safety, Institute of Quality Standards & Testing Technology for Agro-Products, Chinese Academy of Agricultural Sciences, Beijing 100081, China; wangqi2021yw@163.com (Q.W.); nkshaohua@163.com (H.S.);; 2 Shimadzu China MS Center, Beijing 100020, China

**Keywords:** forchlorfenuron, mass spectrometry imaging, matrix-assisted laser desorption/ionization, spatial distribution, migration characteristic

## Abstract

Forchlorfenuron is a widely used plant growth regulator to support the pollination and fruit set of oriental melons. It is critical to investigate the spatial distribution and migration characteristics of forchlorfenuron among fruit tissues to understand its metabolism and toxic effects on plants. However, the application of imaging mass spectrometry in pesticides remains challenging due to the usually extremely low residual concentration and the strong interference from plant tissues. In this study, a matrix-assisted laser desorption/ionization mass spectrometry imaging (MALDI-MSI) method was developed for the first time to obtain the dynamic images of forchlorfenuron in oriental melon. A quantitative assessment has also been performed for MALDI-MSI to characterize the time-dependent permeation and degradation sites of forchlorfenuron in oriental melon. The majority of forchlorfenuron was detected in the exocarp and mesocarp regions of oriental melon and decreased within two days after application. The degradation rate obtained by MALDI-MSI in this study was comparable to that obtained by HPLC-MS/MS, indicating that the methodology and quantification approach based on the MALDI-MSI was reliable and practicable for pesticide degradation study. These results provide an important scientific basis for the assessment of the potential risks and effects of forchlorfenuron on oriental melons.

## 1. Introduction

Forchlorfenuron, a synthetic plant growth regulator, has been used in many horticulture plants to support pollination and increase productivity during the past 30 years [1,2]. Recently, due to its detection in different agro-products and toxicity, forchlorfenuron has received greater scientific and regulatory scrutiny. Forchlorfenuron has been associated with severe hydrometra in the uterus and pathological changes in the ovaries of Sprague–Dawley (SD) rats [3] and classified as a category 2 carcinogenic agent by the European Food Safety Authority [4,5,6]. Adverse effects on fruit quality have also been reported after forchlorfenuron application [7,8].

Investigating the spatial distribution of forchlorfenuron among fruit tissues is important for understanding its metabolism and toxic effects on plants. Some studies have been conducted on kiwifruit, grape, and apple, and the ^14^C-forchlorfenuron exhibited different migration characteristics in a variety of fruits [1]. However, the method lacks molecular specificity and relies on radiolabeled tracers [9,10,11]. In recent years, high-performance liquid chromatography–mass spectrometry (HPLC-MS) has also been reported as an alternative method to study the spatial distribution of forchlorfenuron [12], whereas the necessary tissue homogenates procedure for determination can still not to achieve the in situ analysis of forchlorfenuron in the sample tissue. To better understand the spatial distribution and migration characteristic of forchlorfenuron in fruit, visual information is more useful than chromatographic data. It provides an anatomical distribution of forchlorfenuron inside the fruit.

Mass spectrometry imaging (MSI) coupled with matrix-assisted laser desorption/ionization (MALDI) has proven to be a promising technique for spatial analysis with its high molecular specificity and visualization for endogenous metabolites and exogenous medicines [13,14,15]. However, analyzing pesticides in plant tissues is not easy due to the trace residual concentration and the strong interference from the MALDI matrix [16,17]. It is well-known that in MALDI-MSI, the matrix and its coating pattern can have a significant influence on ionization efficiency in the MALDI ion source, and an internal standard can control for the irreproducibility of ion signals from scan to scan for small-molecule compounds. Therefore, the matrix selection and its coating pattern are the critical steps to obtain high-quality MALDI mass spectrometry (MS) signals for several small-molecule compounds, including sulfonylurea herbicides and fungicide metalaxyl in recent years [18,19,20,21].

Oriental melon (*Cucumis melo var. makuwa*) is an important fruit worldwide, and more than 28 million tons were produced in 2020 (FAOSTAT 2020). In China, more than 42.1% of oriental melons are treated with forchlorfenuron to promote fruit set and increase fruit weight [22]. To the best of our knowledge, no investigation of the spatial distribution of forchlorfenuron in oriental melons has been reported. In this study, we developed an MALDI-MSI method to study the spatial distribution and time-dependent migration of forchlorfenuron in oriental melon fruit. Considering the effective factors on the sensitivity, the matrix and matrix coating pattern were optimized. The isotope internal standard was applied to improve the quantitative capabilities of MSI. This is the first report about the dynamic images of forchlorfenuron in oriental melon and we hope that it will direct future studies on the fate of pesticides in fruit tissues.

## 2. Material and Methods

### 2.1. Chemicals and Reagents

The standard of forchlorfenuron (purity > 98%) was purchased from Dr. Ehrenstorfer (Augsburg, Germany). Forchlorfenuron-d5, the isotopic internal standard (IS), was purchased from Toronto Research Chemicals (Toronto, ON, Canada). HPLC-grade methanol, formic acid, and pure water were obtained from Fisher (Marshalltown, IA, USA). MALDI-grade α-cyano-4-hydroxycinnamic acid (CHCA), 2,5-dihydroxybenzoic acid (DHB), and 9-aminoacridine (9-AA) were purchased from Sigma-Aldrich (St. Louis, MO, USA). Other reagents and solvents used in the present study were of analytical grade.

### 2.2. Preparation of Standard Solutions and Matrix Solutions

The stock solution of 100 mg/L forchlorfenuron standard was prepared in MeOH and diluted to the needed concentrations before use. For the matrix deposition on the sample sections, DHB and 9-AA were prepared in 10 mg/mL in MeOH/water (70/30, *v*/*v*). In total, 50 mg/mL of CHCA was prepared in MeOH/water (70/30, *v*/*v*).

### 2.3. Field Trials and Sample Collection

The field trial was conducted on oriental melons (*Cucumis melo var. makuwa*) from July to October 2018 in a greenhouse at the Chinese Academy of Agricultural Sciences. The field trials were divided into a treatment group and a control group. The dose of forchlorfenuron soluble concentrate (SL) was set to 20 mg/L according to the recommended dose on the registered label. In the treatment group, 20 mg/L forchlorfenuron solution was used to dip the flower and fruit ovary for 1–2 s during the flowering stage with BBCH code 61 of the oriental melon. A separate plot for the no-forchlorfenuron application was used as a control, and oriental melons required manual pollination to generate homozygous parental lines. At least 6 representative oriental melon fruit samples were collected randomly from each plot at 2 h, 1 d, 3 d, and 4 d after pollination or application of forchlorfenuron. All samples were flash-frozen in liquid nitrogen and stored at −80 °C until analysis.

### 2.4. Tissue Sectioning and Matrix Deposition for MALDI-TOF-MSI

Frozen oriental melon samples were cut longitudinally into 35 μm sections and thaw-mounted on indium tin oxide glass slides for MSI at −18 °C using a cryostat microtome (Leica CM1950, Nussloch, Germany). Matrix coating modes were optimized by comparing the signal/noise (S/N) of forchlorfenuron produced under 3 possible matrix coating procedures as follows: (1) Sublimation: 300 mg CHCA was applied by vacuum sublimation using the iMLayer system (Sanyu Electron, Tokyo, Japan) for 8 min at 250 °C. (2) Airbrushing: 500 μL CHCA matrix solution (10 mg/mL, in MeOH and distilled water (*v*/*v*, 7:3) was sprayed onto the tissue surface by airbrush. (MR. Linear Compressor L7/PS270 Airbrush, Tokyo, Japan). The distance between the airbrush tip and the tissue surface was about 8 cm. For the first 3 cycles, the matrix was airbrushed for 2 s at 60 s intervals and, in the following 7 cycles, the matrix was continuously sprayed for 1 s at 30 s intervals. After spraying, the glass slide was air-dried for 5 min to vaporize the solvent. (3) A “two step matrix application” method was used: sublimation combined with an airbrushing step. The first step was the same as that described in the above “(1) Sublimation” part. For the second step, 1 mL matrix (10 mg/mL) solution or methanol was sprayed onto the glass slide for 10 cycles using the airbrush according to the procedures described in “(2) Airbrushing”. Finally, the sample sections were air-dried for 2 min to vaporize the solvent.

### 2.5. Mass Spectrometry Imaging Conditions

Mass spectrometry imaging was performed using a mass microscope (iMScope TRIO, Shimadzu, kyoto, Japan) equipped with a high-resolution optical microscope and a hybrid ion trap time-of-flight (IT-TOF) mass spectrometer with an atmospheric pressure MALDI with a diode-pumped 355 nm Nd: YAG laser (Shimadzu Corporation, Kyoto, Japan). Optical images and ion distribution data under the same microscope were obtained. The laser power was 45 eV and 55 eV for the precursor ion (*m*/*z* 248.05) and the product ions (*m*/*z* 129.02, *m*/*z* 155.00) of forchlorfenuron, respectively. The mass spectrometry conditions for the precursor ion (MS stage 1) and the product ion (MS stage 2) of forchlorfenuron were as follows:

MS stage 1: sample voltage, 3.5 kV; detector voltage, 1.95 kV; number of laser shots, 80; repetition rate, 1000 Hz; laser diameter, 2 (20 μm); ion polarity, positive; mass range, 200–300; laser intensity, 45 eV.

MS stage 2: sample voltage, 3.5 kV; detector voltage, 1.95 kV; number of laser shots, 80; repetition rate, 1000 Hz; laser diameter, 2 (20 μm); precursor ion, *m*/*z* 248.056; mass range, 50–300; laser intensity, 55 eV.

### 2.6. Quantitative Analysis

Standard curve: A series of forchlorfenuron standard solutions (0.1, 0.5, 1.0, 5.0, 10.0, 20.0 mg/L) containing 5 mg/L forchlorfenuron-d5 isotopic IS solution were deposited onto the blank oriental melon sections before matrix application using a micropipettor (1 μL); each concentration was repeated three times. The standard curve equation was plotted using the ion intensity extracted from the regions of the standard point of Qual Browser to Microsoft Excel.

Sample quantification: Methods of internal standard application were adapted from Chumbley et al. [23] All oriental melon tissue sections received IS (5 mg/L forchlorfenuron-d5 isotopic IS solution, 1μL) in different regions (*n* = 9) before the sublimation of the matrix. The extracted ion currents for forchlorfenuron (*m*/*z* 248.05→129.02) and forchlorfenuron-d5 (*m*/*z* 253.05→129.02) were plotted using data extracted from the regions of interest (ROI) of oriental melon sections to Microsoft Excel.

### 2.7. Statistical Analysis

The data acquisition, visualization, and quantification were performed by the imaging MS Solution Version 1.30 Software (Shimadzu Corporation, Tokyo, Japan). Regions of interest (ROI) were manually defined in the analysis software using the optical and MSI data image. The data are expressed as the mean value of three replicates with the standard deviation (SD).

## 3. Results and Discussion

### 3.1. Optimization of Matrix

**Figure 1:**
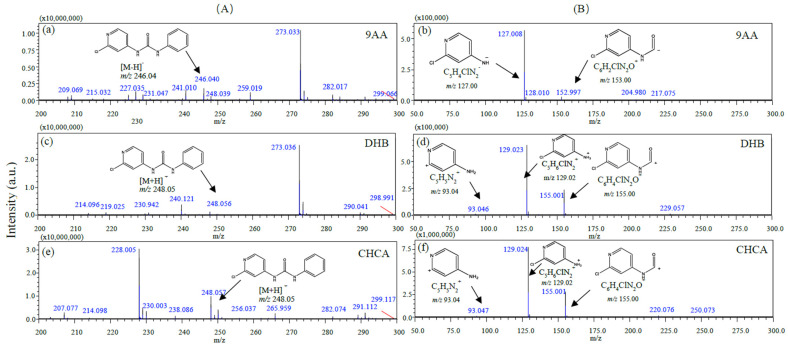
The structures and mass spectra of forchlorfenuron by MALDI-MSI with different matrices, the forchlorfenuron standard solution (1 µL, 100 mg/L) deposited on blank oriental melon sections (2 h after manual pollination) before matrix sublimation. (**A**) Precursor ion mass spectrum. (**B**) Product ion mass spectrum. (**a**,**b**) 9AA in the negative mode. (**c**,**d**) DHB in the positive mode. (**e**,**f**) CHCA in the positive mode.

Based on the fact that the matrix is one of the most important characteristics influencing sensitivity in MALDI-MSI, the effects of three matrices (DHB, CHCA, and 9-AA) on the ionization and sensitivity of forchlorfenuron were investigated. As shown in Figure 1, CHCA produced the highest signal responses of the target precursor ion (*m*/*z* 248.05) and product ions (*m*/*z* 129.02, *m*/*z* 155.00) of forchlorfenuron, which were 8–15 times higher than those obtained by using 9AA or DHB. 

**Figure 2:**
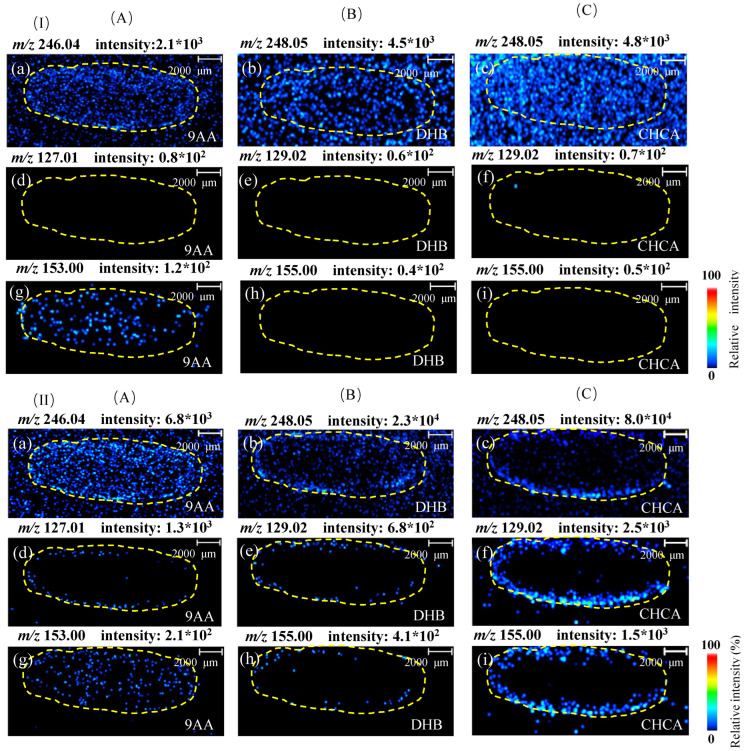
Representative ion images of forchlorfenuron in oriental melon sections in control group (**I**) and treatment group (**II**) by MAIDL-MSI with different matrices. (**A**) 9AA in the positive mode. (**a**) precursor ion. (**d**) product ion 1. (**g**) product ion 2. (**B**) DHB in positive ion mode. (**b**) Precursor ion. (**e**) product ion 1. (**h**) product ion 2. (**C**) CHCA in positive ion mode. (**c**) Precursor ion. (**f**) product ion 1. (**i**) product ion 2.

In addition, the background interference of these matrices in MSI analysis was evaluated by sublimating these three matrices on the oriental melon sections from the control group and treatment group (2 h after manual pollination or application of the forchlorfenuron), respectively. As shown in Figure 2I, the interfering signals were obviously observed at all precursor ions (*m*/*z* 248.05, Figure 2Ia–c) and product ions (*m*/*z* 155.00, Figure 2Ig) using the DHB matrix. However, compared with control groups (Figure 2I), the relative intensity and profiles of the signals of forchlorfenuron were significantly increased and clearly observed in treatment oriental melon sections (Figure 2II). Moreover, the CHCA matrix showed a higher signal intensity, provided the clear visualization of forchlorfenuron, and avoided background interferences from matrix and melon tissues in the images for product ions (*m*/*z* 129.02 and *m*/*z* 155.00). These results suggested the high selectivity of the CHCA matrix for forchlorfenuron detection in tissue sections by MALDI-MSI. Furthermore, with less background and high sensitivity, the secondary product ions (*m*/*z* 129.02 and *m*/*z* 155.00) of forchlorfenuron were selected for subsequent analysis.

### 3.2. Optimization of Matrix Application Methods

**Figure 3:**
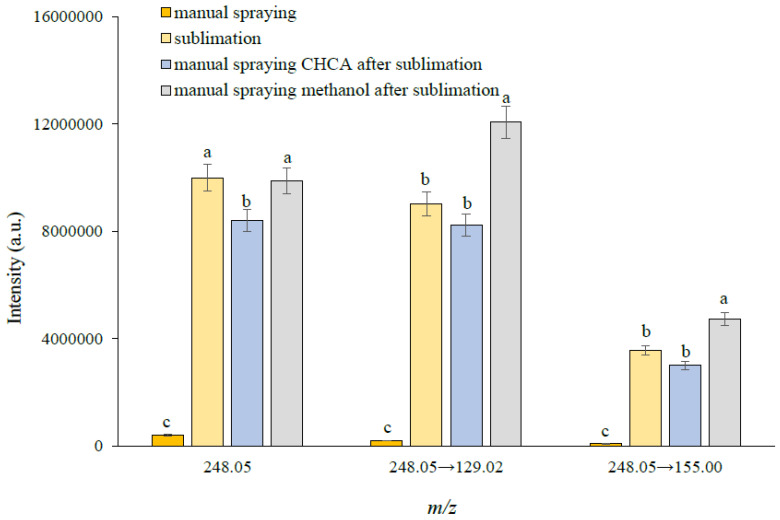
Comparison of detection sensitivities of forchlorfenuron after different matrix coating modes; forchlorfenuron standard solution (1 µL, 100 mg/L) was deposited on the blank oriental melon section (*n* = 3). Bars that do not share similar letters denote statistically significant differences (*p* < 0.05) determined by a pairwise *t*-test with Bonferroni-adjusted *p*-values.

The matrix preparation and deposition procedure are the other critical parts of a successful MALDI-IMS analysis [24,25]. In this study, in order to obtain the maximum ion signal intensity and good-quality images of forchlorfenuron, different matrix coating modes such as matrix sublimation and manual spraying were adopted for the pretreatment of the tissue sections containing 100 mg/L of forchlorfenuron. As shown in Figure 3, compared to the manual spraying of CHCA, the sublimation CHCA method provided higher signal intensities of forchlorfenuron, suggesting that this method produced relatively small crystallization and homogeneous matrix–forchlorfenuron cocrystal, which was similar to the results reported in the previous paper [14,16,18,26]. In addition, an attempt to further improve the sensitivity of forchlorfenuron was made by the “two-step matrix application”, which combined matrix sublimation and the airbrushing method used in this study. When CHCA was used in the second step by airbrushing, it was found that there was a decrease in the precursor ion and the two product ions in comparison to the one-step method, which was possibly associated with the uneven distribution and larger CHCA matrix crystal on the oriental melon tissue. However, when methanol was used in the second step by airbrushing, as expected, it produced a better sensitivity than that used in CHCA. It was possibly related to the increase in the rate of matrix–analyte co-crystallization by spraying methanol, which was similar to the results reported in an analysis of octreotide in the mouse target tissue [27]. Therefore, the two-step matrix coating method, combining the sublimation of CHCA and manual spraying of methanol, was adopted in the subsequent study.

### 3.3. Method Validation

**Figure 4:**
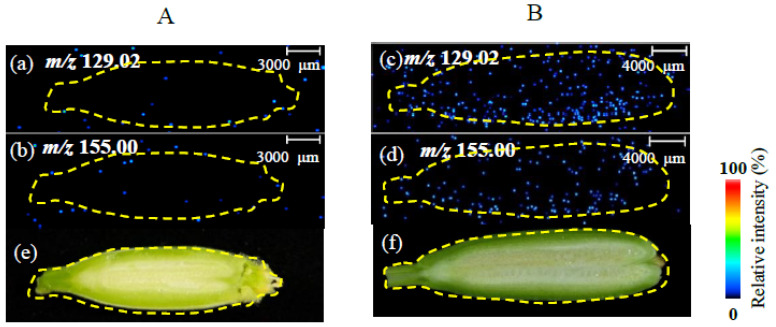
Distribution of forchlorfenuron in oriental melon, as shown by iMScope, in (**A**) control group (2 d after chasmogamy) and (**B**) treatment group (2 d after application of forchlorfenuron). (**a**–**d**) Product ion mass imaging MS of *m*/*z* 129.02, 155.00. (**e**,**f**) Optical image of oriental melon slides.

To validate the developed MSI analytical method, the spatial distribution of forchlorfenuron was examined in oriental melon fruit from the control group (2 d after chasmogamy) and treatment group (2 d after application of forchlorfenuron) in field trials. In the control group (Figure 4A), forchlorfenuron was scarcely detected, as illustrated by the imaging of both secondary product ions. In the application group (Figure 4B), the profiles of the signals of product ions (*m*/*z* 129.02 and *m*/*z* 155.00) of forchlorfenuron were clearly distinguished from the background noise, and the intensities of the product ions (*m*/*z* 129.02 and *m*/*z* 155.00) of forchlorfenuron were 2.5 × 10^3^ and 1.5 × 10^3^, respectively. This result indicated that the optimized method could be used to investigate the spatial distribution of forchlorfenuron in melon fruits.

### 3.4. Quantitative Determination of Forchlorfenuron in Oriental Melon by MSI

**Figure 5:**
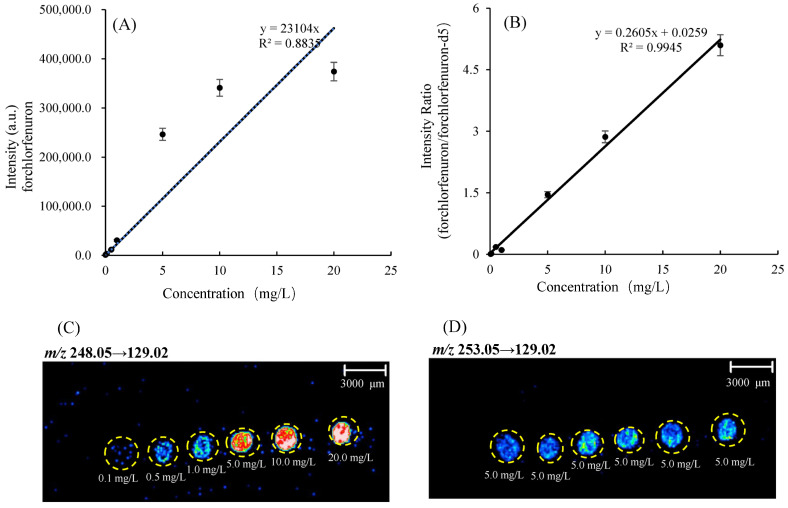
MALDI-MSI experiment for quantitative analysis of forchlorfenuron from oriental melon tissue. Calibration curve (*n* = 3) of forchlorfenuron generated using (**A**) the average intensity of *m*/*z* 129.02 and (**B**) the ratio average intensity of *m*/*z* 248.05→129.02/253.05→129.02. (**C**) Ion intensity of *m*/*z* 248.05→129.02 of forchlorfenuron was used to generate MS/MS images. Calibration spots increase in concentration from 0.10 to 20.0 mg/L. (**D**) Ion intensity of *m*/*z* 253.05→129.02 of d5-forchlorfenuron (5.0 mg/L) was used to generate MS/MS images.

Figure 5A showed a calibration curve by plotting the average intensity of forchlorfenuron against the concentrations on oriental melon sections. There was a positive linear correlation between signal intensity and the concentration of forchlorfenuron between 0.10 mg/L and 20.0 mg/L (*n* = 3), with a linear correlation coefficient (R^2^) of 0.8198. The intensity ratio of forchlorfenuron/forchlorfenuron-d5 against the concentrations on oriental melon sections was shown in Figure 5B. The linear correlation coefficient (R^2^) was expected to improve to 0.9945 by normalizing the forchlorfenuron/forchlorfenuron-d5 ion signal ratio for quantitative analysis, and the relative standard deviations of the measurements from each spot in the tissue section were less than 13.0%. Linearity and scan-to-scan reproducibility were significantly improved when normalizing by the IS. These improvements can be attributed to the control for ionization variability from the matrix and tissue heterogeneity, inefficient analyte extraction, and ionization suppression effects in tissue surfaces [28].

Furthermore, as shown in Figure 5C, the imaging bright changes in spotted forchlorfenuron solutions also matched well with the forchlorfenuron standard concentrations from 0.10 mg/L to 20.0 mg/L and maintained stability in the d5-forchlorfenuron solutions of constant concentration (Figure 5D). Therefore, MALDI-MSI was shown to be suitable for the quantitative analysis of forchlorfenuron in oriental melon after internal calibration. Multiple analyses of replicate tissue sections from multiple melons produced reproducible quantitative results. Therefore, MSI was shown to be suitable for the quantitative analysis of forchlorfenuron in oriental melon, and the LOQ was found to be 0.1 mg/kg.

### 3.5. Spatial Distribution and Migration Characteristics of Forchlorfenuron in Oriental Melon by MALDI-MSI

**Figure 6:**
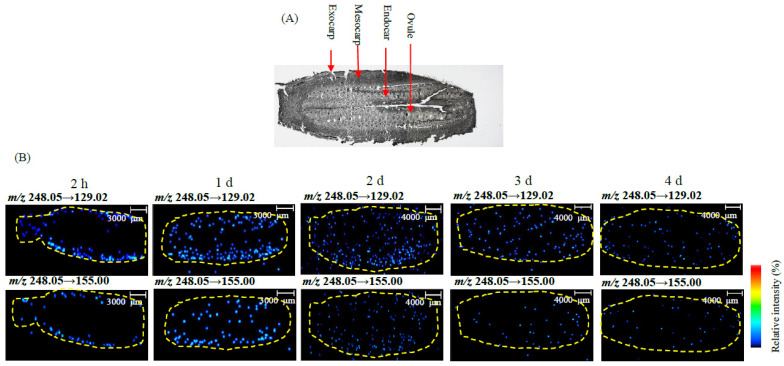
In situ identification and imaging of forchlorfenuron in oriental melon after forchlorfenuron application, as shown by iMScope. (**A**) Optical image of oriental melon sections acquired by microscope via 1.25× magnification. (**B**) Representative ion images of forchlorfenuron in oriental melon at treatment group (2 h–4 d after application of forchlorfenuron).

The developed MALDI-MSI method was used to investigate the spatial distribution and time-dependent permeation of forchlorfenuron in oriental melon fruit in field trails. Figure 6A shows the optical image of a longitudinal cross section of an oriental melon fruit under the microscope of the iMScope instrument.

As shown in Figure 6B, a gradual and continuous migration and degradation of forchlorfenuron in oriental melon occurred between 2 h and 4 days after application. It was found that forchlorfenuron was mainly distributed in the exocarp region of oriental melon fruit, and the concentration was estimated at 0.8 mg/kg after 2 h. After one day, forchlorfenuron generally migrated from the exocarp into the mesocarp region with a concentration of 0.4 mg/kg. After two days, most forchlorfenuron gradually migrated from the mesocarp into the endocarp region with a concentration of 0.1 mg/kg, indicating that forchlorfenuron degraded quickly and penetrated in oriental melon. After four days, no distinct signal was observed in the imaging of the forchlorfenuron ions observed, which means that the concentrations of forchlorfenuron were lower than 0.1 mg/kg. It is maybe related to the metabolism of 4-hydroxyphenyl-forchlorfenuron in oriental melon fruit [29]. The degradation rate obtained by MALDI-MSI in this study was comparable to that obtained by HPLC-MS/MS in our previous study [29]. In our previous paper, the half-life of forchlorfenuron in oriental melon fruit was 1.29 days, and 4-hydroxyphenyl-forchlorfenuron was first detected at 4d with concentrations of 4.5 μg/kg by HPLC-MS/MS.

The radioactive residues of ^14^C-forchlorfenuron were major in the skin fractions of kiwifruit after 127 days, while approximately 62% of the radioactive residues were associated with the apple pulp and skin after 114 days. In our study, the majority of forchlorfenuron was detected in the exocarp and mesocarp regions of oriental melon and decreased within two days after application. These results may be related to the increasing flesh thickness of oriental melon post-harvest after forchlorfenuron treatment [30]. These results can help us better understand the effect and biological fate of forchlorfenuron in oriental melon.

## 4. Conclusions

In conclusion, an MALDI-MSI method was developed for the first time to obtain the dynamic images of forchlorfenuron in oriental melon. The matrix and matrix coating methods were optimized to improve the sensitivity of forchlorfenuron in melon tissue greatly. Good quantitative accuracy and sharp images were obtained when plotting characteristic fragment ions normalized with IS. The method was successfully applied to investigate the spatial distribution and time-dependent permeation of forchlorfenuron in oriental melon fruit after forchlorfenuron treatment. Most forchlorfenuron was detected in the exocarp and mesocarp regions within two days after application, and the overall concentration gradually decreased over time. The degradation rate obtained by MALDI-MSI in this study was comparable to that obtained by HPLC-MS/MS, which provides an important scientific basis for assessing the potential risks to consumers and effects of forchlorfenuron on oriental melons.

## Data Availability

The data used to support the findings of this study can be made available by the corresponding author upon request.
